# Gonadotropin-Releasing Hormone Agonists Sensitize, and Resensitize, Prostate Cancer Cells to Docetaxel in a p53-Dependent Manner

**DOI:** 10.1371/journal.pone.0093713

**Published:** 2014-04-10

**Authors:** Roberta M. Moretti, Marina Montagnani Marelli, Deanne M. Taylor, Paolo G. V. Martini, Monica Marzagalli, Patrizia Limonta

**Affiliations:** 1 Dipartimento di Scienze Farmacologiche e Biomolecolari, Università degli Studi di Milano, Milano, Italy; 2 Department of Obstetrics, Gynecology, and Reproductive Sciences, Division of Reproductive Sciences, UMDNJ-Robert Wood Johnson Medical School, Morristown, New Jersey, United States of America; 3 Drug Discovery and Translational Research, Shire Rare Diseases Unit, Lexington, Massachusetts, United States of America; II Università di Napoli, Italy

## Abstract

Gonadotropin-releasing hormone (GnRH) receptors are expressed in prostate cancer, specifically in the most aggressive stage of the tumor (castration-resistant prostate cancer, CRPC) for which the standard treatment, docetaxel-based chemotherapy, can only improve the median survival time by few months. We previously showed that GnRH agonists exert an antitumor activity in CRPC cells; however, a link between GnRH receptors and the apoptotic machinery remains to be defined. Aim of this study was to evaluate whether, in CRPC cells, GnRH agonists might affect the expression/activity of apoptosis-related proteins and might sensitize, or resensitize, cancer cells to chemotherapeutics. We demonstrated that, in p53-positive DU145 cells, GnRH agonists: a) increase the expression of the proapoptotic protein Bax; this effect is mediated by the phosphorylation (activation) of p53, triggered by the p38 MAPK; b) potentiate the antiproliferative/proapoptotic activity of docetaxel; c) resensitize docetaxel-resistant cells to the antitumor activity of the cytotoxic drug. These data indicate that GnRH agonists sensitize and, more importantly, resensitize DU145 CRPC cells to chemotherapy in a p53-dependent manner. To confirm the crucial role of p53 in the activity of GnRH agonists, experiments were performed in p53-null PC3 cells. We found that GnRH agonists fail to increase Bax expression and do not potentiate the cytotoxic activity of docetaxel. These results may provide a rationale for novel combination treatment strategies, especially for docetaxel-resistant CRPC patients expressing a functional p53 protein.

## Introduction

Prostate cancer is the most commonly diagnosed cancer for men and the second leading cause of cancer-related deaths among men in Western Countries [1]. Most prostate cancers are dependent on the presence of androgens for growth and survival, and androgen ablation therapy, aimed to block androgen secretion/activity, represents the most effective initial treatment [2], [3]. This therapy includes surgical or chemical castration, achieved by: administration of gonadotropin-releasing hormone (GnRH) analogs; blocking of the binding of androgens to their receptor by antiandrogens; inhibition of steroidogenic enzymes. Unfortunately, despite an excellent initial response, in approximately 2 to 3 years, most prostate cancers will progress to castration-resistant prostate cancer (CRPC) stage with increased proliferation and malignancy [4], [5]. For CRPC patients, taxane-based chemotherapy represents the treatment of choice [6], [7]. Docetaxel acts by binding to tubulin to promote polymerization and prevents microtubule depolymerization in the absence of guanosine triphosphate. It has also been shown to induce tumor cell death by affecting the expression/activity of multiple cancer-specific targets, including downregulation of the antiapoptotic protein Bcl-2 and upregulation of the proapoptotic protein Bax [8], [9]. However, despite the initial demonstration of a better survival with docetaxel-based chemotherapy, the improvement was found to be only a progression-free survival of few months [6], [10]. Thus, treatment of patients with CRPC that progresses after docetaxel-based chemotherapy remains a significant clinical challenge. The identification of novel strategies aimed at overcoming docetaxel resistance will likely improve the therapeutic options for these patients.

GnRH was first identified as the hypothalamic key regulator of the reproductive functions. By binding to specific receptors (GnRH-R) on pituitary gonadotropes GnRH activates the pituitary-gonadal axis. GnRH agonists, when given continuously and at high doses, desensitize pituitary GnRH-R, thus suppressing gonadal steroid secretion; on the basis of their activity, these compounds represent the most widely and successfully utilized medical treatment for androgen-responsive prostate cancer [2], [11].

It is now well established that GnRH receptors are expressed in prostate cancer cells, specifically in CRPC cells and tissues [12]–[15].

These receptors (as well as GnRH receptors in breast and gynecological cancer cells and tissues) have been first characterized in terms of binding affinity. However, contrasting results have been reported: one class of low-affinity binding sites [12], [13], [16]–[18]; two types of receptors (one with high affinity and one with low affinity) [19]–[21]; one single class of high affinity GnRH binding sites [22]–[25]. In particular, we reported the presence of low affinity GnRH receptors in prostate cancer cells [12], [13]. The reason for this discrepancy is still a matter of debate; however, it might be due to the different experimental conditions adopted (different cancer cell lines and ligands, evaluation of the binding affinity in cancer cells/tissues expressing the binding sites *vs*. cells engineered to overexpress the receptors, cell-specific posttranslational modifications of the receptor, *etc.*).

These initial contrasting observations stimulated the characterization of cancer GnRH receptors at the molecular level. Specifically, we reported that both the mRNA coding for the human pituitary GnRH receptor and the corresponding protein are expressed in prostate cancer cells, either androgen-dependent or castration-resistant [26]. Moreover, the nucleotide sequence of the cDNA coding for the tumor receptors has been shown to correspond to that previously reported for the pituitary receptors [27], [28].

We further showed that GnRH agonists, through activation of locally expressed GnRH receptors, exert a strong antiproliferative effect on prostate cancer cells, both *in vitro* and *in vivo* [12], [29], [30]; this antitumor activity is specific since it is completely abrogated after the silencing of the GnRH receptor by means of a specific siRNA [31].

In addition to reduced cell proliferation, apoptosis has been suggested to be involved in the antitumor activity of GnRH analogs. However, the data so far available on this issue are still controversial [30], [32]–[35].

Here, we confirm our previous observation that GnRH agonists do not, by themselves, induce apoptosis in CRPC cells. However, in p53-expressing DU145 prostate cancer cells, they increase the expression of the proapoptotic protein Bax, through phosphorylation at Ser-15 (*i.e*., activation) of p53, the major regulator of the intrinsic apoptotic pathway; activation of this p53-Bax apoptotic signaling is triggered by p38 MAPK phosphorylation. We also show that GnRH agonists sensitize DU145 cells to the antimitotic activity of docetaxel. More importantly, GnRH agonists resensitize docetaxel-resistant DU145 cells to the death-inducing activity of the chemotherapeutic agent. These data indicate that, in p53-positive prostate cancer cells, targeting locally expressed GnRH receptors by means of GnRH agonists sensitizes and resensitizes cancer cells to chemotherapy, in a p53-dependent manner. The crucial role played by p53 is further demonstrated by the observation that GnRH agonists do not affect Bax expression and fail to potentiate the apoptotic activity of docetaxel in p53-null PC3 prostate cancer cells. Taken together, these results represent the rationale for combination treatment strategies for docetaxel-resistant CRPC patients, expressing a functional p53 protein.

## Materials and Methods

### Cell Cultures

The human castration-resistant DU145 (p53-positive) and PC3 (p53-null) prostate cancer cell lines were purchased from the American Tissue Culture Collection. Both PC3 and DU145 cells represent the most appropriate and widely utilized model of CRPC in the literature [36]–[40]. Cells were routinely grown in Roswell Park Memorial Institute-1640 (RPMI-1640) medium supplemented with 5% Fetal Bovine Serum (FBS), glutamine (1 mmol/L) and antibiotics (100 IU/mL penicillin G sodium and 100 μg/mL streptomycin sulfate), in humidified atmosphere of 5% CO_2_/95% air at 37°C.

### Materials and Antibodies

The GnRH agonist Goserelin acetate [D-Ser(tBu)^6^Aza-Gly^10^-GnRH, Zoladex, GnRH-A] was kindly provided by AstraZeneca Pharmaceuticals. The GnRH antagonist Antide (Ant) and docetaxel (Doc) were purchased from Sigma-Aldrich.

In all the experiments, the GnRH agonist has been utilized at the dose of 10^−6 ^mol/L, on the basis of previous studies, from the authors’ laboratory as well as from others, aimed to investigate the molecular aspects of the antitumor activity of GnRH analogs in CRPC cells [29], [41]–[46]. The same range of doses were also utilized to investigate the antitumor activity of GnRH agonists in several experimental models of cancer cells overexpressing the GnRH receptor [16], [47]–[49].

Pifithrin-α, the specific inhibitor of p53 transcriptional activity, and SB203580, the specific p38 MAPK inhibitor, were purchased from Santa Cruz Biotechnology and from Sigma-Aldrich, respectively.

Antibodies used for Western blotting experiments: rabbit anti-human Bax (1∶500; #2772), rabbit anti-human p38 MAPK (1∶1,000;. #9212) and rabbit anti-human p-p38 MAPK (1∶1,000; #9211) from Cell Signaling Technology; mouse monoclonal anti-human Bcl-2 (1∶250; #Sc-7382), mouse monoclonal anti-human p53 (1∶1,000; #Sc-53394), rabbit anti-human p-p53 (Ser-15; 1∶1,000; #Sc-101762) and goat anti-human actin 1–19 (1∶1,000; #Sc-1616) from Santa Cruz Biotechnology.

### Microarray Analysis

DU145 prostate cancer cells were seeded (5×10^5^ cells/dish) in 10-cm tissue culture dishes; after 48 hours, cells were treated with GnRH-A (10^−6 ^mol/L) for 24 hours and total RNA was prepared with the use of the RNeasy mini kit (Qiagen), according to the instructions of the manufacturer. After quality control using a bioanalyzer (Agilent 2100), RNAs were labeled according to Affymetrix protocol using One Cycle Labeling Kit (Affymetrix). 15 μg of resulting cRNAs were hybridized onto whole genome microarray U133 plus 2.0. After hybridization on Affymetrix HG-U133 Plus 2.0 chips, gene expression values were estimated for each probe set using packages in the Bioconductor suite [50]. Genes were normalized and analyzed with the Robust Multichip Analysis (RMA) method within the affy package [51] and the GCRMA package [52]. The differences in log expression levels for both RMA and GCRMA normalized data were evaluated by the two-tailed t-test as implemented in the limma package [53]. Genes with *P*-values<0.05 and the absolute expression fold change greater than 1.5 were considered as significantly differentially expressed (DE) between treated and untreated cells. A gene list was generated by taking the overlap of DE genes generated by limma from both the RMA and GCRMA normalization methods.

### Western Blot Analysis

At the end of the experiments, cells were washed with PBS and lysed in RIPA buffer (0.05 mol/L Tris.HCl pH 7.7, 0.15 mol/L NaCl, 0.8% SDS, 10 mmol/L EDTA, 100 μmol/L NaVO4, 50 mmol/L NaF, 0.3 mmol/L PMSF, 5 mmol/L iodoacetic acid) containing leupeptin (50 μg/mL), aprotinin (5 μl/mL) and pepstatin (50 μg/mL). Protein concentration was determined using the BCA method. Protein extracts (30 μg) were resuspended in Sample buffer (0.5 mol/L Tris.HCl pH 6.8, 20% glycerol, 10% SDS, 0.2% 2β-mercaptoethanol, 0.05% blue bromophenol) and heated at 95°C for 5 minutes. Following electrophoretic separation by SDS-PAGE, proteins were transferred onto nitrocellulose membranes. Membranes were blocked in 3% non fat dry milk prior to incubation at room temperature for 2 hours with the specific primary antibodies at the appropriate dilutions. Detection was done using a horseradish-peroxidase-conjugated secondary antibody and enhanced chemiluminescence reagents (Supersignal Chemiluminescence Detection System). In each Western blot experiment actin expression was evaluated as a loading control. For each protein analysis, three different experiments were done; the densitometric analysis reported in the figures was performed on the results obtained from the three different experiments.

### Cell Proliferation and Cell Viability Studies

DU145 and PC3 cells were seeded (1×10^4^ cells per dish) in 6-cm dishes. After 2 days, cells were treated with GnRH-A (10^−6 ^mol/L) for 24 hours, either alone or in the presence of the GnRH antagonist Antide (Ant, 10^−6 ^mol/L), followed by docetaxel (10 nmol/L) for 48–72 hours. Cells treated with each of the two drugs alone or without any treatment served as controls. At the end of the treatments, cells were harvested and counted by hemocytometer. For viability studies, DU145 and PC3 cells were treated as described and cell viability was measured by Trypan Blue exclusion assay. The number of dead cells was measured by counting Trypan Blue staining cells.

### Caspase-3 Enzyme Activity Assay

Caspase-3-like activity was assessed using the CaspACE colorimetric assay kit (Promega). DU145 cells were seeded (2×10^5^ cells/dish) in 10-cm dishes. After 2 days, cells were pretreated with GnRH-A (10^−6 ^mol/L) for 24 hours and then treated with docetaxel (10 nmol/L) for 72 hours. At the end of the treatments, cells (both adherent and detached) were lysed in the lysis buffer contained in the kit followed by centrifugation (15,000×*g* for 10 minutes at 4°C). Caspase-3-like activity was assessed by following the proteolytic cleavage of the colorimetric substrate Ac-DEVD-pNA. Samples were read at 405 nm in a spectrophotometer using a 100 μL quarz cuvette. The pan-caspase inhibitor z-VAD-fmk was used to confirm assay specificity.

### Colony Formation Assay

For the development of docetaxel-resistant cells (DU145-R), DU145 cells seeded in 10-cm dishes were serially treated with docetaxel (10 nmol/L, once a week) until they developed the ability to grow and divide in the presence of the drug (8 weeks). To investigate whether GnRH agonists might resensitize DU145-R cells to the activity of docetaxel, a standard clonogenic assay was performed. DU145-R cells were seeded at 1,000 cells/well in six-well plates and allowed to attach for 24 hours. Cells were then treated with GnRH-A (10^−6 ^mol/L) for 24 hours followed by docetaxel (10 nmol/L) for 72 hours. At the end of the treatment, cells were rinsed and fresh medium was added. Cells were cultured for 14 days in 5% FBS containing medium and then fixed and stained with crystal violet. Images of stained colonies were captured by a Nikon photocamera.

### Statistical Analysis

When appropriate, data were analyzed by Bonferroni’s test, after one-way analysis of variance. *P*­values<0.05 were considered significant.

## Results

### GnRH Agonists Increase Bax Expression in DU145 Cells

**Figure 1:**
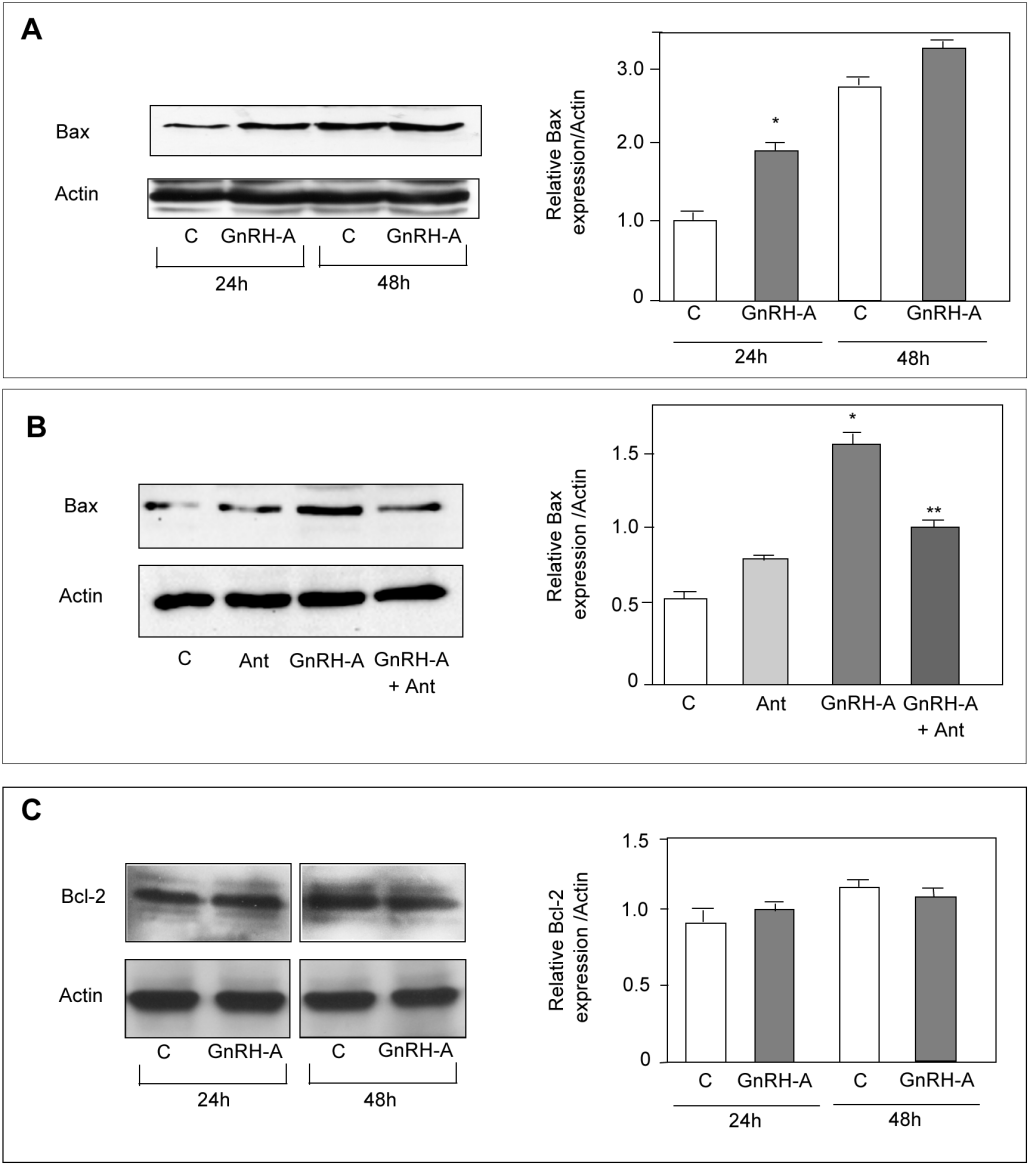
In DU145 cells, GnRH agonists increase the expression of the proapoptotic protein Bax, without affecting the expression of the antiapoptotic protein Bcl-2. (A) DU145 cells were treated with GnRH-A (10^−6 ^mol/L) for either 24 or 48 hours. Western blotting was performed on whole cell extracts by using an anti-Bax antibody. As expected, GnRH-A treatment increases Bax protein expression at 24 hours of treatment. (B) DU145 cells were treated with GnRH-A (10^−6 ^mol/L) and with the GnRH antagonist Antide (Ant, 10^−6 ^mol/L), either alone or in combination, for 24 hours. Western blotting was performed as described in A). Ant, given alone, does not affect Bax expression, while GnRH-A, as expected, increases the expression of Bax. This stimulatory effect of GnRH-A is specific since it is abrogated by the cotreatment of the cells with the GnRH antagonist. (C) Treatment of DU145 cells with GnRH-A (10^−6 ^mol/L) for either 24 or 48 hours, does not affect Bcl-2 expression, as evaluated by Western blotting. For both Bax and Bcl-2 protein expression analysis one representative of three different experiments is shown. Data represent means ± SEM. *P<0.05 *versus* C (untreated controls); **P<0.05 *versus* GnRH-A-treated cells.

We previously reported that GnRH agonists exert an antiproliferative effect on DU145 prostate cancer cells. However, it is still unclear whether apoptosis might also be involved in the antitumor activity of these compounds [30], [32]–[35]. The BCL-2 family of cell death regulators (both proapoptotic and prosurvival) represents a critical control point in the intrinsic pathway of apoptosis. The balance of these two classes of proteins determines the fate of the cell. The proapoptotic protein Bax, through oligomerization with Bak, translocates from the cytoplasm to mitochondria to increase mitochondrial outer membrane permeabilization, triggering cytochrome *c* release and caspase-3 activity [54]. Here, we investigated whether GnRH agonists might affect the expression of genes involved in the apoptotic pathway. DU145 cells were treated with GnRH-A for 24 hours and changes in gene expression profile were evaluated by genome-wide transcriptomic analysis. Among the genes whose expression was modified by the treatment we focused our attention on the expression of *Bax*. *Bax* expression was significantly increased by 1.67 fold (Table 1); this increase was confirmed at the protein level by Western blotting (Fig. 1A). The stimulatory effect of GnRH-A on Bax expression was found to be specific since it was abrogated by the cotreatment of the cells with the GnRH antagonist Antide (Fig. 1B). On the other hand, the GnRH agonist did not affect the expression of the antiapoptotic protein Bcl-2 (Fig. 1C).

**Table 1:** Affymetrix Human Genome U133 Plus Array.

Probe	Gene	Description	Fold change
211833_s	*BAX*	*BCL2-associated X protein*	1,67348

DU145 cells were treated with GnRH-A (10^−6^ M) for 24 hours. Changes in gene expression profile were evaluated by genome wide transcriptomic analysis and statistically analyzed as described in Materials and Methods.

### In DU145 Cells GnRH Agonists Upregulate Bax Expression through the p53 Signaling Pathway

**Figure 2:**
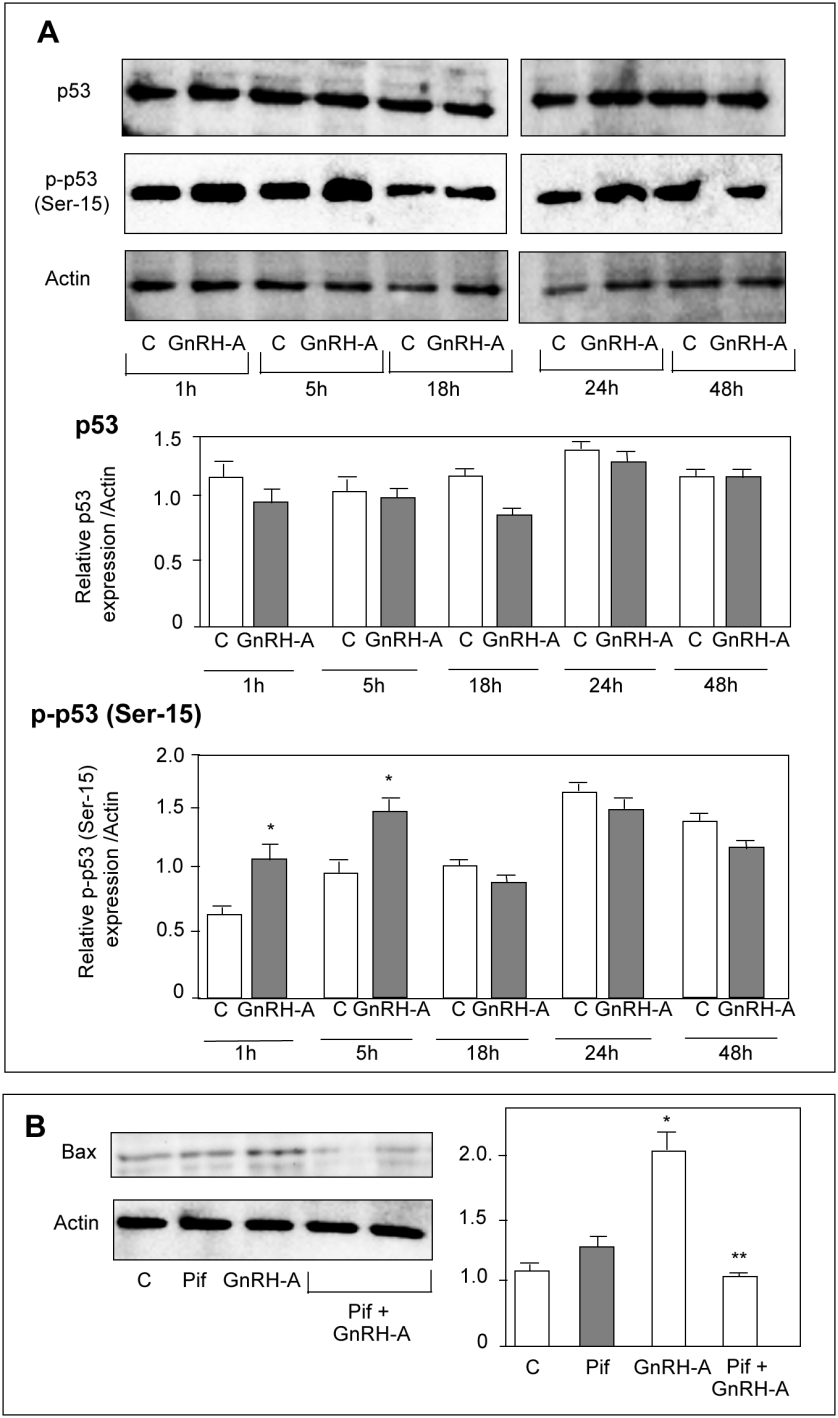
In DU145 cells, GnRH agonists increase the expression of Bax through p53 phosphorylation (Ser-15). (**A**) DU145 cells were treated with GnRH-A (10^−6 ^mol/L) for different time intervals (1–48 hours). Western blot analysis was performed on whole cell extracts by using p53 or p-p53 (Ser-15) antibodies. Treatment with GnRH-A does not affect the expression of p53 at any time intervals considered; however, the levels of the serine-15 phosphorylated form of the protein are significantly increased after 1 and 5 hours of treatment. (**B**) DU145 cells were treated with GnRH-A (10^−6 ^mol/L) either alone or after a pretreatment with pifithrin-α (Pif, 30 μmol/L, for 2 hours), the specific p53 inhibitor. Western blot analysis was performed on whole cell extracts using Bax antiobody. The results obtained show that pifitrhin-α, when given alone, does not affect Bax expression. As expected, GnRH-A increases the expression of Bax; this effect is completely abolished when the cells are pretreated with pifithrin-α. One representative of three different experiments, for each of the analyses performed, is shown. Data represent means ± SEM. *P<0.05 *versus* C (untreated controls); **P<0.05 *versus* GnRH-A-treated cells.

**Figure 3:**
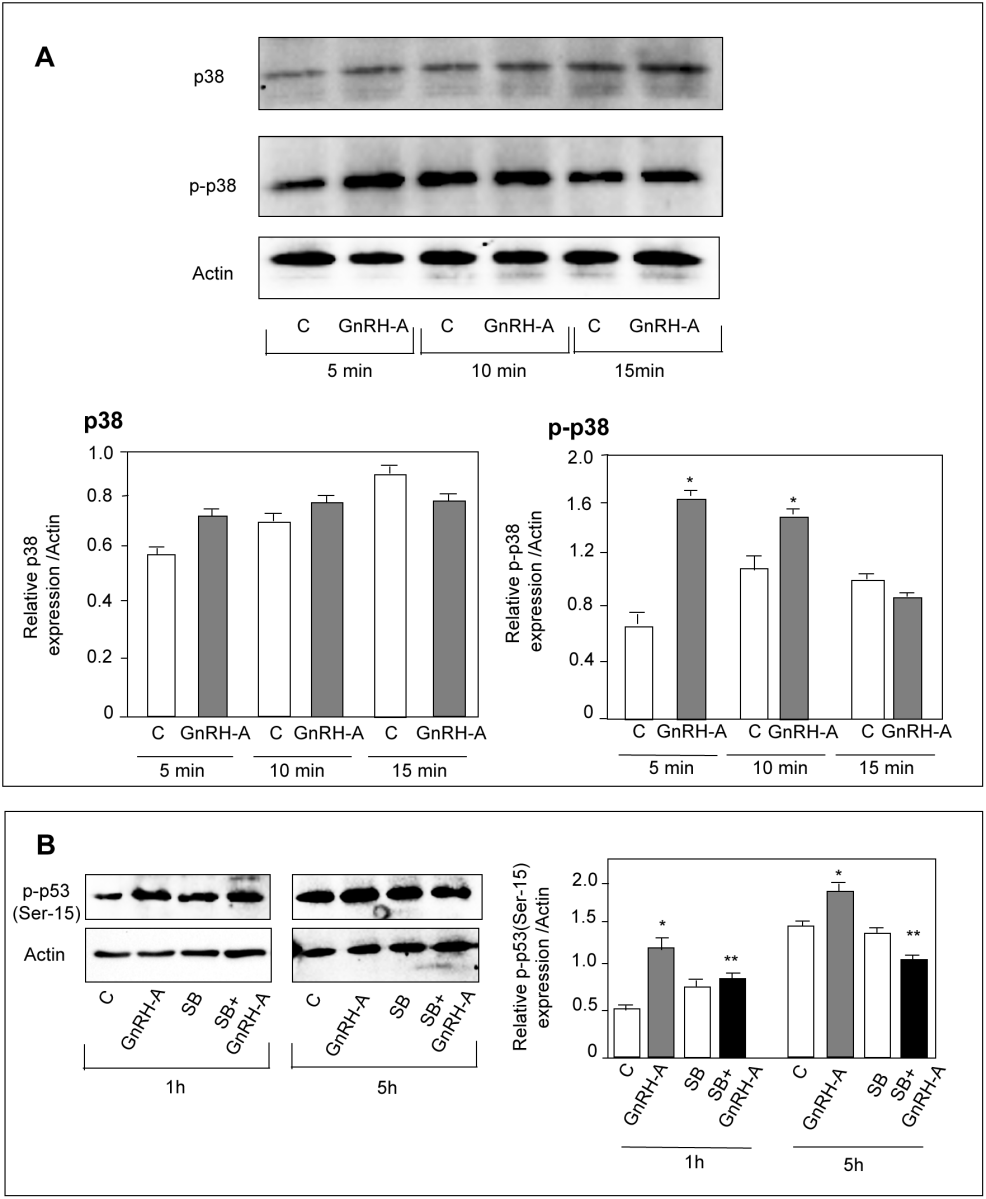
In DU145 cells, GnRH agonists trigger p53 phosphorylation (Ser-15) through activation of p38 MAPK. (**A**) DU145 cells were treated with GnRH-A (10^−6 ^mol/L) for different time intervals (5–15 minutes). Western blot analysis was performed on whole cell extracts by using p38 and p-p38 antibodies. Treatment with GnRH-A does not affect the expression of p38 at any time interval considered. On the other hand, GnRH-A significantly increases the levels of the phosphorylated form of p38 (p-p38) at 5 and 10 minutes of treatment.(**B**) As expected, GnRH-A increases the expression levels of p-p53, confirming previous results; densitometric analysis of the data demonstrate that this effect is significantly reduced when the cells are pretreated with the specific p38 inhibitor SB203580 (SB, 1 μmol/L, for 30 minutes). One representative of three different experiments, for each of the analyses performed, is shown. Data represent means ± SEM. *P<0.05 *versus* C (untreated controls); **P<0.05 *versus* GnRH-A-treated cells.

The key role of p53 in the intrinsic apoptotic pathway is well established [55]. After being activated through phosphorylation at Ser-15, p53 translocates into the nucleus to regulate the expression of proapoptotic genes, such as *Bax* [56]. Moreover, p53 phosphorylation was reported to be dependent on the activity of the p38 MAPK [57]. By Western blot analysis we found that, in DU145 cells, GnRH-A treatment (1–48 hours) did not affect the expression of p53; however, the levels of the serine-15 phosphorylated (*i.e*., active) form of the protein were significantly increased after 1 and 5 hours of treatment (Fig. 2A). When the cells were pretreated (2 hours) with pifithrin-α (the p53 inhibitor) the stimulatory effect of GnRH-A (24 hours) on Bax expression was completely abolished (Fig. 2B). We also found that treatment of the cells with GnRH-A (5–15 minutes) did not affect the expression level of p38 MAPK; however it significantly increased the levels of the phosphorylated form of the MAPK, at 5 and 10 minute time intervals (Fig. 3A). Pretreatment (30 minutes) of the cells with SB203580 (1 μmol/L), the specific p38 inhibitor, significantly reduced the stimulatory effects of GnRH-A on p53 phosphorylation (1 and 5 hours) (Fig. 3B). Thus, in DU145 cells GnRH agonists upregulate the expression of the proapoptotic protein Bax through p53 phoshorylation; p38 MAPK is an upstream activator of this p53-Bax signaling pathway.

### GnRH Agonists Sensitize p53-positive DU145 Cells to the Cytotoxic Activity of Docetaxel

**Figure 4:**
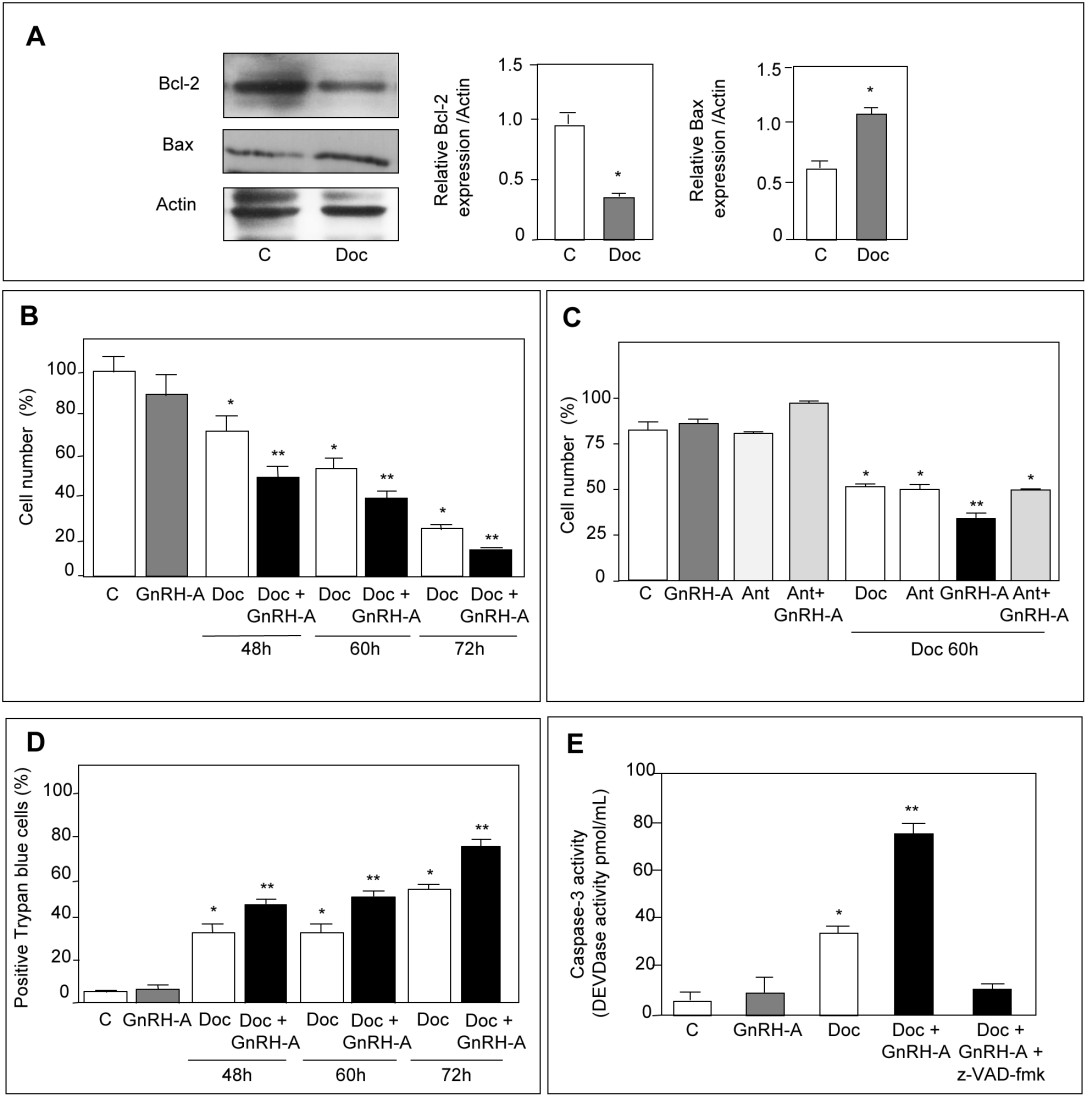
GnRH agonists sensitize p53-positive DU145 cells to the antiproliferative/proapoptotic activity of docetaxel. (**A**) Western blot analysis was performed to confirm that the cytotoxic activity of docetaxel (Doc) is mediated by a perturbation of the ratio pro-to-antiapoptotic proteins. DU145 cells were treated with docetaxel (10 nmol/L) for 48 hours. As expected, docetaxel decreases the expression of the antiapoptotic protein Bcl-2 while increasing Bax expression. One representative of three different experiments is shown. (**B**) DU145 cells were pretreated with GnRH-A (10^−6 ^mol/L) for 24 and then with docetaxel (10 nmol/L) for different time intervals (48–72 hours). At the end of the treatments, cells were counted by hemocytometer. Pretreatment of the cells with GnRH-A significantly increases the antiproliferative effect of docetaxel at all time intervals considered. (**C**) DU145 cells were pretreated with GnRH-A (10^−6 ^mol/L) and with the GnRH antagonist Antide (Ant, 10^−6 ^mol/L), either alone or in combination, for 24 hours, and then with docetaxel (10 nmol/L) for 60 hours. At the end of the treatments, cells were counted by hemocytometer. Pretreatment of the cells with GnRH-A significantly increases the antiproliferative effect of docetaxel; this effect is specific since it is completely abrogated by the cotreatment of the cells with Ant. (**D**) At the end of the treatments (performed as described in B), cell viability was measured by Trypan Blue exclusion assay. The number of dead cells was measured by counting Trypan Blue staining cells. Data are expressed as percent of stained cells/total cells. Docetaxel significantly increases the number of dead cells; at all time intervals, this effect is significantly potentiated by pretreatment of the cells with GnRH-A. (**E**) DU145 cells were pretreated with GnRH-A for 24 hours and then treated with docetaxel for 72 hours. At the end of the treatment caspase 3-like activity was assessed using the CaspACE colorimetric assay kit. Pretreatment of the cells with GnRH-A significantly potentiates the proapototic effect of docetaxel in terms of caspase-3 activity. The effect is specific since it is counteracted by the simultaneous treatment with the pan-caspase inhibitor z-VAD-fmk. In B-E each experimental group consisted of six replicates and each experiment was repeated three times. Data represent means ± SEM. *P<0.05 *versus* C (untreated controls); **P<0.05 *versus* docetaxel-treated cells.

A perturbation in the balance between pro- and antiapoptotic factors is a crucial step in the cell decision to undergo apoptosis or survival. Based on the stimulatory effect of GnRH agonists on Bax expression, mediated by p53 activation, we investigated whether GnRH agonists might induce apoptosis in DU145 cells. By FACS analysis we couldn’t observe any proapoptotic effect of GnRH-A on these cells (data not shown). These observations were not unexpected since it was previously reported that, in CRPC cells, GnRH agonists reduce cell proliferation without inducing apoptosis [30], [32], [35]. The reason why, in DU145 cells, GnRH agonists increase Bax levels without triggering the apoptotic event is intriguing. However, it is well known that DU145 cells overexpress the antiapoptotic protein Bcl-2, and the levels of this protein are not affected by the GnRH-A treatment (see Fig. 1C). Thus, it is possible that, in these cells, the increased expression of Bax induced by GnRH-A is not sufficient to efficiently enhance the Bax/Bcl-2 ratio to trigger apoptosis. On the basis of these considerations, we reasoned that GnRH agonists, through upregulation of proapoptotic factors, might sensitize prostate cancer cells to the activity of cytotoxic drugs, known to act by increasing the expression of proapoptotic factors while decreasing that of antiapoptotic proteins. We focused our attention on docetaxel since: a) it represents the treatment of choice for CRPC [4], [5]; b) it has been shown to induce tumor cell death through upregulation of the proapoptotic protein Bax and downregulation of the antiapoptotic protein Bcl-2 [8], [9]. First, by Western blotting, we confirmed that docetaxel (48 hours) significantly decreases the expression of Bcl-2, while increasing that of Bax (Fig. 4A). Then, we evaluated whether GnRH agonists might potentiate the antitumor activity of docetaxel. We first found that, when given alone, GnRH-A (24 hours) does not affect the proliferation of DU145 cells. This was not unexpected, since we have previously reported that, in CRPC cells, GnRH agonists can exert a significant antiproliferative effect only when the treatments are performed for longer time intervals (4–7 days) [29]. Similar data have been reported for other types of tumors [58], [59]. Then we demonstrated that pretreating DU145 cells with GnRH-A (24 hours) significantly improves the antiproliferative effects of docetaxel (48–72 hours) at all time intervals (Fig. 4B). Moreover, we could show that the sensitizing effect of GnRH-A to docetaxel was specific since it was completely abrogated by the cotreatment of the cells with the GnRH antagonist Antide (Fig. 4C). In the same experimental conditions, assessment of cell viability was performed by Trypan Blue exclusion assay. GnRH-A, when given alone, did not affect cell viability (Fig. 4D). As expected, docetaxel significantly increased the number of positive Trypan Blue stained cells (dead cells). This effect was significantly potentiated by pretreatment of the cells with GnRH-A at all time intervals (Fig. 4D). To confirm that the pretreatment with GnRH agonists sensitizes CRPC cells to the antitumor activity of docetaxel, DU145 cells were treated with GnRH-A (24 hours) and then with the cytotoxic drug for 72 hours. Caspase-3 activity was then evaluated as a marker of apoptotic cell death. GnRH-A, given alone, did not affect caspase-3 activity; as expected, docetaxel increased enzymatic activity. Pretreatment of the cells with GnRH-A significantly potentiated the proapototic effect of the chemotherapeutic agent. The effect is specific since it was counteracted by the simultaneous treatment of the cells with the pan-caspase inhibitor z-VAD-fmk (Fig. 4E).

These results indicate that, in p53-positive DU145 prostate cancer cells GnRH agonists specifically sensitize the cells to the cytotoxic activity of docetaxel; this effect is likely the consequence of the GnRH-A-induced activation of the p38/p53/Bax apoptosis signaling pathway.

### GnRH Agonists Resensitize p53-positive DU145 Cells to the Cytotoxic Activity of Docetaxel

**Figure 5:**
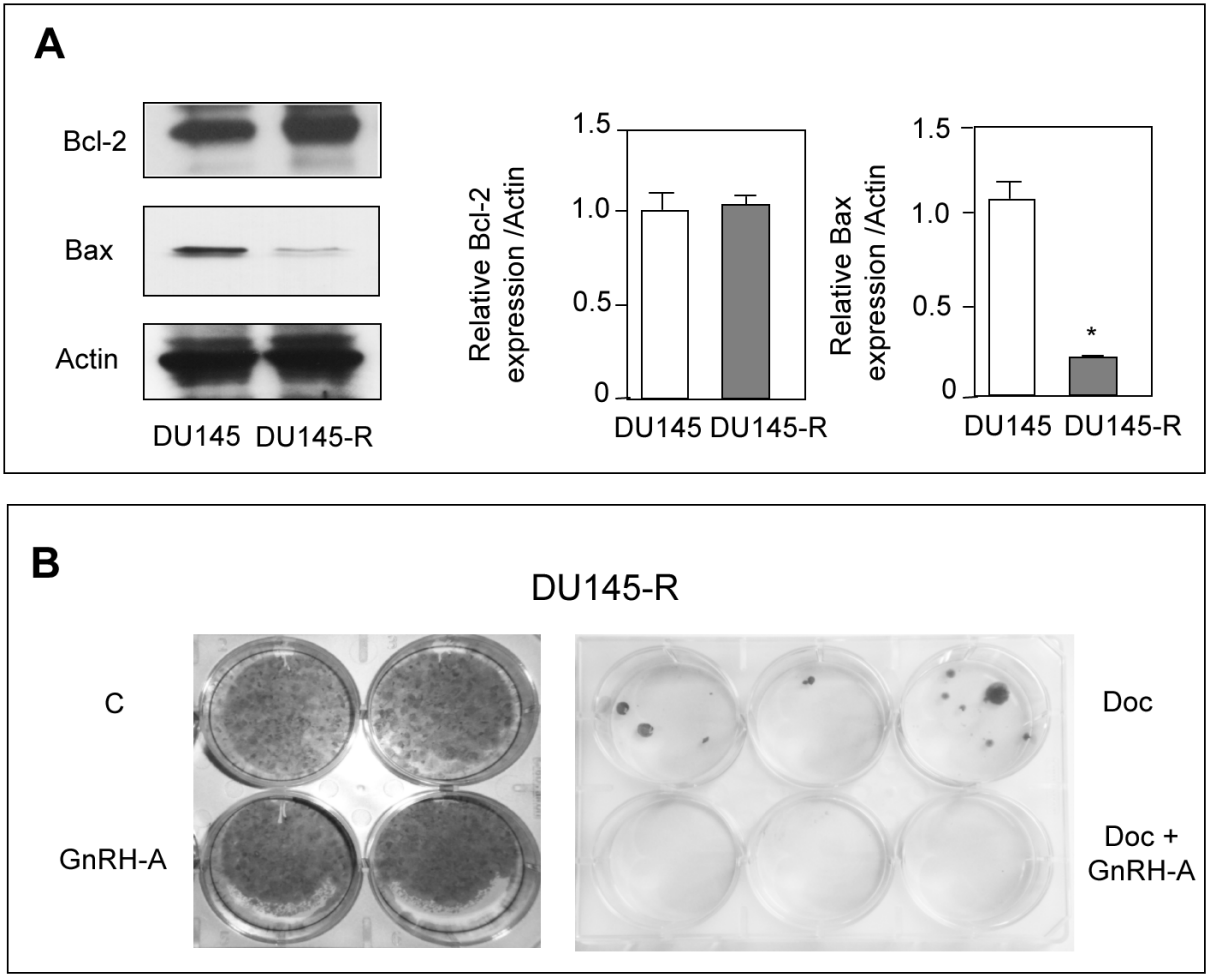
GnRH agonists resensitize p53-positive DU145 cells to the cytotoxic activity of docetaxel. DU145 prostate cancer cells were made resistant to docetaxel (Doc) after treatment with the chemotherapeutic drug (10 nmol/L) once a week for 8 cycles. (**A**) Western blot analysis of Bcl-2 and Bax in docetaxel-resistant (DU145-R) cells. In DU145-R cells the expression of the proapoptotic protein Bax looks significantly down-regulated, while the expression of the antiapoptotic protein Bcl-2 is not significantly modulated. One representative of three different experiments is shown. Data represent means ± SEM. *P<0.05 *versus* DU145 cells. (**B**) Clonogenic survival assay of DU145-R cells pretreated with GnRH-A (10^−6 ^mol/L) for 24 hours and then treated with docetaxel (10 nmol/L) for 72 hours. GnRH-A given alone does not affect the ability of DU145-R cells to form colonies. DU145-R cells can form colonies in the presence of the chemotherapeutic agent, confirming their acquisition of resistance to the drug. Combination treatment completely abolishes the colony formation ability of DU145-R cells. Each experimental group consisted of two/three replicates and each experiment was repeated three times with identical results.

To obtain docetaxel-resistant cells (DU145-R), DU145 cells were serially treated with docetaxel (once a week for 8 weeks) until they developed the ability to grow in the presence of the drug. In these cells, the resistance to the cytotoxic compound was associated with a significant decrease of Bax levels, without any change in the level of expression of Bcl-2 (Fig. 5A), confirming previous observations reporting little consensus between taxane-resistance and overexpression of Bcl-2 [60]. On the other hand, our data clearly indicate that DU145 cells can contrast and overcome the antitumor activity of chemotherapeutic drugs by increasing the ratio between anti- and proapoptotic proteins. By clonogenic assay, we then investigated whether GnRH agonists might resensitize docetaxel-resistant prostate cancer cells to the proapoptotic activity of the chemotherapeutic drug. Treatment of DU145-R cells with GnRH-A (24 hours) alone did not affect the ability of the cells to form colonies (Fig. 5B). As expected, DU145-R cells formed colonies in the presence of docetaxel (72 hours), confirming their acquisition of resistance to the drug. However, combination treatment (GnRH-A for 24 hours followed by docetaxel for 72 hours) completely abolished the colony formation ability of DU145-R cells (Fig. 5B). These results indicate that GnRH agonists can resensitize docetaxel-resistant prostate cancer cells to the antitumor activity of the chemotherapeutic drug.

### GnRH Agonists do not Sensitize p53-null PC3 Cells to the Cytotoxic Activity of Docetaxel

**Figure 6:**
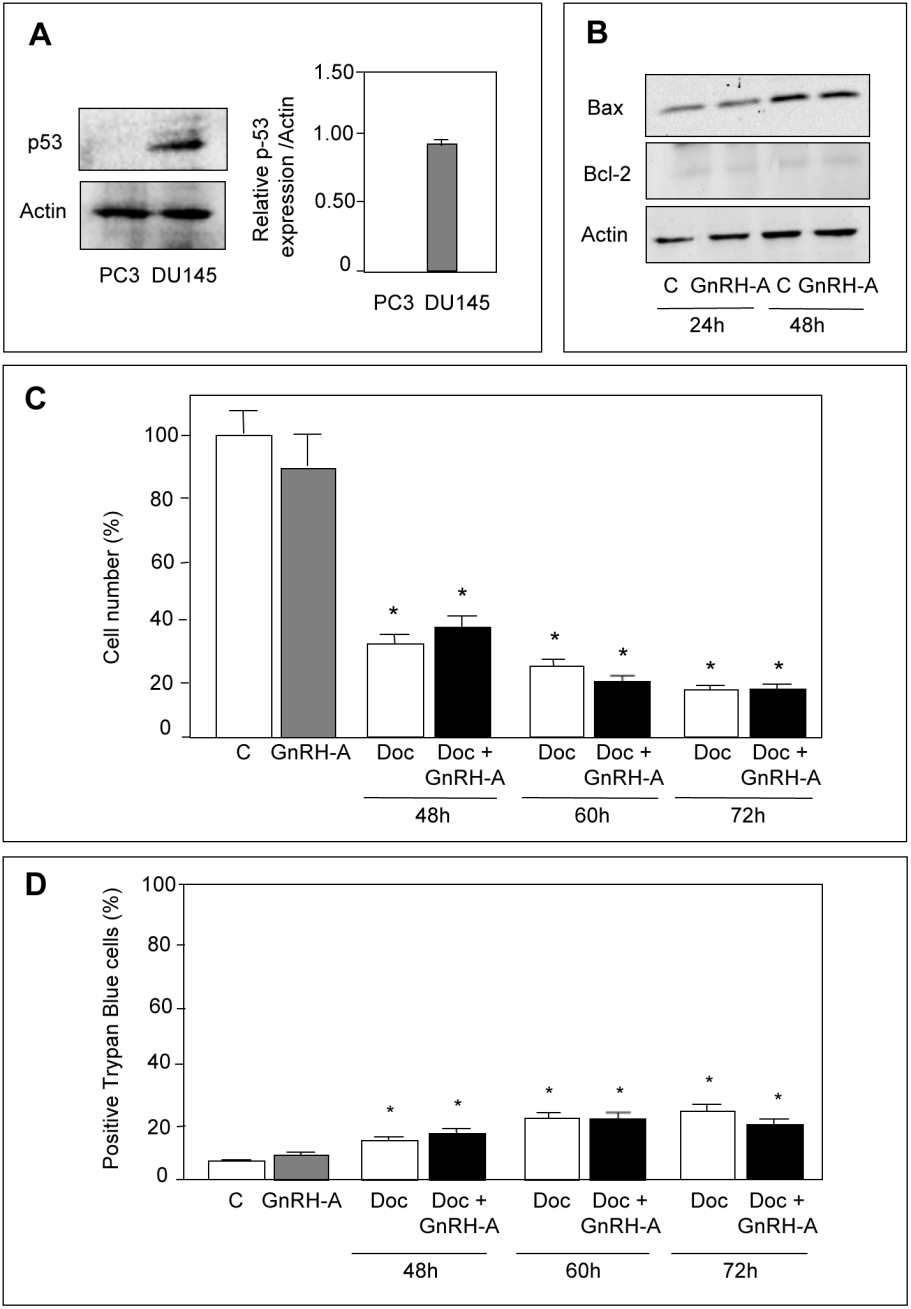
GnRH agonists do not sensitize p53-null PC3 cells to the antiproliferative/proapoptotic activity of docetaxel. (**A**) Western blot analysis was performed to confirm the absence of p53 expression in PC3 cells. It is shown that p53 is expressed in DU145 cells while it is absent in PC3 cells. (**B**) Western blot analysis performed on whole cells extracts shows that treatment of PC3 cells with GnRH-A (10^−6 ^mol/L, for 24–48 hours) does not affect the expression of either the proapoptotic (Bax) or the antiapoptotic (Bcl-2) protein. One representative of three different experiments is shown. (**C**) PC3 cells were pretreated with GnRH-A (10^−6 ^mol/L) for 24 and then with docetaxel (10 nmol/L) for different time intervals (48–72 hours). Cells were then counted by hemocytometer. Pretreatment of the cells with GnRH-A does not potentiate the antiproliferative effect of docetaxel at all time intervals considered. (**D**) At the end of the treatments (as described in C), cell viability was measured by Trypan Blue exclusion assay. The number of dead cells was measured by counting Trypan Blue staining cells. Data are expressed as percent of stained cells/total cells. Docetaxel significantly increases the number of dead cells; pretreatment of the cells with GnRH-A does not potentiate the cytotoxic activity of docetaxel at any time interval considered. Each experimental group consisted of six replicates and each experiment was repeated three times. Data represent means ± SEM. *P<0.05 *versus* C (untreated controls).

To confirm the crucial role of p53 in the ability of GnRH agonists to sensitize prostate cancer cells to the cytotoxic activity of docetaxel, experiments were performed in p53-null PC3 prostate cancer cells [61]. By Western blotting we could confirm that p53 is not expressed in PC3 cells (Fig. 6A). Moreover, treatment of the cells with GnRH-A (24 or 48 hours) did not affect either Bax or Bcl-2 protein levels (Fig. 6B). As expected, pretreatment of the cells with GnRH-A (24 hours) did not potentiate the antiproliferative (Fig. 6C) and the cytotoxic (Fig. 6D) activity of docetaxel (48–72 hours).

These results confirm that the ability of GnRH agonists to sensitize prostate cancer cells to cytotoxic therapy is dependent on a functional p53 protein.

## Discussion

The therapeutic options for castration-resistant prostate cancer patients are still very limited [4], [5]. Docetaxel is considered the treatment of choice for these men; however, the response to the drug is often associated with increased resistance to apoptosis and progression of the tumor with a very short overall survival [6], [10]. For this reason, great efforts are now being made to define new molecular therapies aimed at potentiating the effectiveness of current cytostatic drugs and/or at overcoming chemotherapy-resistance.

It is well established that GnRH receptors are expressed in prostate cancer cells, specifically in CRPC cells and tissues [12]–[15].

GnRH agonists, through activation of these receptors, exert a strong antitumor effect (antiproliferative, antimetastatic), by interfering with the activity of mitogenic growth factors [30], [62]. However, it is still unclear whether, in addition to reduced cell proliferation, apoptosis might be involved in the antitumor activity of these compounds.

In this paper, we first investigated whether activation of GnRH receptors might affect the expression of genes/proteins involved in the apoptotic pathway. We studied CRPC DU145 prostate cancer cells because they have been shown to respond to GnRH agonists and express p53. We found that, in DU145 cells, GnRH-A significantly increased the expression of the proapoptotic protein Bax (both at the mRNA and at the protein level). Moreover, these compounds induced the phosphorylation (*i.e.,* activation) of the key proapoptotic protein p53 at Ser-15, through the activity of the p38 MAPK. More importantly, we could demonstrate that blocking the activity of p53 (by means of the specific inhibitor pifithrin-α) completely abolished the stimulatory effects of GnRH-A on Bax expression. These results indicate that, in DU145 prostate cancer cells, GnRH agonists upregulate the expression of the proapoptotic protein Bax; this effect is dependent on the activation of p53, by p38 MAPK.

It has been previously shown that DU145 cells express p53 with two point mutations at residues 223 and 274 located in the DNA binding domain of the protein [63]. In these cells, p53 lacks the antioncogenic activity of the wild-type protein. In the present paper we demonstrated that GnRH-A induced p53 phosphorylation at Ser-15 and this was associated with increased Bax expression, suggesting that serine phosphorylation of mutant p53 restored the activity of the protein. These observations are in line with those reported by Lin and coworkers [64], showing that, in DU145 cells, resveratrol promotes Ser-15 phosphorylation of p53 increasing its binding to DNA. According to these authors, it is also possible that treatment of DU145 cells with proapoptotic signals might promote expression of a wild-type allele of p53 in sufficient abundance to be functional [64]. More recently, Volate and coworkers [65] demonstrated that the phytochemical gossypol induces apoptosis in DU145 cells through phosphorylation of p53 at Ser-392 and caspases 9 and 3 activation, further confirming that the mutated form of p53 can restore its activity after being phosphorylated. It is also interesting to remind that two-thirds of CRPC patients have wild-type p-53 in their tumors [66].

The observation that GnRH agonists promote Bax expression, through p53 activation, prompted us to investigate whether GnRH agonists might induce apoptosis in DU145 cells. However, by FACS analysis we couldn’t detect any proapoptotic effect of GnRH-A on these cells, confirming previous observations [30], [32], [35]. On the basis of these results, we reasoned that GnRH agonists, through Bax upregulation, might promote a perturbation in the balance between pro- and antiapoptotic factors, thus sensitizing prostate cancer cells to the activity of cytotoxic drugs, such as docetaxel. To verify this hypothesis, we first confirmed that in DU145 cells docetaxel exerts its proapoptotic activity by increasing the expression of Bax while decreasing that of Bcl-2, confirming previous observations [8], [9]. Then, we could demonstrate that a pretreatment of CRPC cells with GnRH-A significantly potentiates the antiproliferative/proapoptotic activity of docetaxel. GnRH agonists, followed by docetaxel when the tumors develop castration resistance, represent the treatment of choice for prostate cancer patients [2]–[7], [67]. Our results support the accepted notion that this treatment strategy may provide a better outcome than orchiectomy followed by docetaxel in this clinical setting. In line with these observations, Gnanapragasam and coworkers [14] have reported that in patients with established progression to hormone refractory disease, GnRH agonist-based therapy, in the presence of high prostate cancer GnRH receptor expression, is associated with improved disease-specific survival. Unfortunately, to date there are no studies investigating the differences in outcomes between prostate cancer patients treated with GnRH analogs followed by docetaxel *vs*. orchiectomy followed by the chemotherapeutic drug.

It is now well established that, after first-line docetaxel-based therapy, most prostate cancer patients develop progressive disease associated with resistance to the cytotoxic drug. Novel tubulin-binding semi-synthetic taxane drugs (*i.e*., cabazitaxel) are now being investigated [68], [69]. However, common adverse events with cabazitaxel, such as neutropenia, lead to the suggestion that this agent should be administered cautiously and with appropriate monitoring [70]. Thus, identification of new second-line regimens for CRPC patients progressing after docetaxel is needed. Interestingly, in this paper we could show, by clonogenic assay, that GnRH agonists, through p53-mediated Bax expression, can resensitize docetaxel-resistant prostate cancer cells, characterized by downregulation of Bax, to the antitumor activity of the chemotherapeutic drug. These data indicate that a combination therapy, based on GnRH agonists followed by docetaxel, might represent an effective treatment strategy for CRPC patients after development of docetaxel-resistance.

Taken together, our results indicate that, in DU145 prostate cancer cells, GnRH agonists increase the expression of the proapoptotic protein Bax, in a p53-dependent manner; by increasing the ratio pro-to-antiapoptotic proteins, GnRH agonists sensitize, and more importantly resensitize, castration-resistant prostate cancer cells to the cytotoxic/proapoptotic activity of docetaxel.

To further confirm the crucial role of p53 in the ability of GnRH agonists to sensitize CRPC cells to chemotherapeutic drugs, we investigated the effects of a pretreatment with GnRH-A on the cytotoxic effects of docetaxel in p53-null PC3 prostate cancer cells. As expected, we found that GnRH-A was unable to potentiate either the antiproliferative or the antiapoptotic activity of the cytotoxic drug.

In conclusion, the observations reported in this paper demonstrate that GnRH agonists can sensitize and, most importantly, resensitize CRPC cells to the cytotoxic activity of chemotherapeutic drugs, in a p53-dependent manner. These results may be translated into the development of novel GnRH analog-based combination therapies, especially for docetaxel-resistant, p53-positive, CRPC patients. To further support the hypothesis of such a novel treatment modality, the results obtained from the in *vitro* studies here reported should be confirmed by *in vivo* experiments performed in nude mice bearing DU145 cell xenografts.

Moreover, these data strongly support the notion that molecular profiling of prostate cancer cells is crucial for the identification of tumor biomarkers predictive of therapeutic response and of disease outcome.
